# Synthesizing qualitative and quantitative evidence on non-financial access barriers: implications for assessment at the district level

**DOI:** 10.1186/s12939-015-0181-z

**Published:** 2015-06-09

**Authors:** Thomas S. O’Connell, K. Juliet A. Bedford, Michael Thiede, Di McIntyre

**Affiliations:** Health Section, Programme Division, UNICEF, New York, USA; Anthrologica, Oxford, UK; School of Anthropology, University of Oxford, Oxford, UK; Scenarium Group GmbH, Berlin, Germany; Health Economics Unit, University of Cape Town, Cape Town, South Africa

**Keywords:** Health system strengthening, Access, Utilization, Equity, UHC, Universal health coverage, Qualitative research, Quantitative research, Non-financial barriers, Health services accessibility

## Abstract

**Introduction:**

A key element of the global drive to universal health coverage is ensuring access to needed health services for everyone, and to pursue this goal in an equitable way. This requires concerted efforts to reduce disparities in access through understanding and acting on barriers facing communities with the lowest utilisation levels. Financial barriers dominate the empirical literature on health service access. Unless the full range of access barriers are investigated, efforts to promote equitable access to health care are unlikely to succeed. This paper therefore focuses on exploring the nature and extent of non-financial access barriers.

**Methods:**

We draw upon two structured literature reviews on barriers to access and utilization of maternal, newborn and child health services in Ghana, Bangladesh, Vietnam and Rwanda. One review analyses access barriers identified in published literature using qualitative research methods; the other in published literature using quantitative analysis of household survey data. We then synthesised the key qualitative and quantitative findings through a conjoint iterative analysis.

**Results:**

Five dominant themes on non-financial access barriers were identified: ethnicity; religion; physical accessibility; decision-making, gender and autonomy; and knowledge, information and education. The analysis highlighted that non-financial factors pose considerable barriers to access, many of which relate to the acceptability dimension of access and are challenging to address. Another key finding is that quantitative research methods, while yielding important findings, are inadequate for understanding non-financial access barriers in sufficient detail to develop effective responses. Qualitative research is critical in filling this gap. The analysis also indicates that the nature of non-financial access barriers vary considerably, not only between countries but also between different communities within individual countries.

**Conclusions:**

To adequately understand access barriers as a basis for developing effective strategies to address them, mixed-methods approaches are required. From an equity perspective, communities with the lowest utilisation levels should be prioritised and the access barriers specific to that community identified. It is, therefore, critical to develop approaches that can be used at the district level to diagnose and act upon access barriers if we are to pursue an equitable path to universal health coverage.

**Electronic supplementary material:**

The online version of this article (doi:10.1186/s12939-015-0181-z) contains supplementary material, which is available to authorized users.

## Introduction

The need for an equitable path to universal health coverage (UHC) has been repeatedly emphasised [1, 2]. A key element of UHC is that of ensuring access to and use of needed health services for everyone. This can only be achieved if existing barriers to access are identified and addressed. The access literature has historically been dominated heavily by a focus on financial barriers, such as the myriad of studies analysing the relationship between health insurance coverage and service use, and on the barriers to access created by user fees [3, 4]. These are important components, but they do not account for the multitude of non-financial obstacles that may impede access, and lead to inequitable access, as well as contribute to high levels of unmet need. Even if financial barriers are reduced, non-financial barriers will remain and undermine efforts to achieve equitable access to health care.

Therefore, in moving towards the access goal of UHC, the full range of barriers should be explored [5–7]. To counterbalance the overwhelming focus on financial access barriers, not only in the literature but also in the policy and management context of seeking ways to overcome access barriers, there is a need to firmly cast the spotlight on non-financial access barriers. This is part of the rationale for this paper.

Another element of the rationale for this paper is that, in our view, researchers, policy-makers and health managers are often overly focused on gaps in service provision including staff and essential commodities [8]. This ignores the contention that access requires bi-directional interaction between health services and their target population [9] and that access can be assessed in terms of the degree of fit between health services and need [10]. Hence, efforts to address access barriers (or ‘lack of fit’) may require not only ‘supply-side’, but also ‘demand-side’ interventions. To develop effective interventions in pursuit of universal access to needed health care, it is necessary to understand in detail both supply- and demand-side constraints to access.

In order to capture the widest range of information possible on non-financial access barriers and both their supply- and demand-side aspects, we undertook structured reviews of the literature presenting quantitative and qualitative empirical research. As the nature of access barriers tend to differ across types of health services, it is advisable to focus on ‘tracer services’. The reviews synthesised evidence on non-financial barriers to access and utilisation of maternal, newborn, and child health (MNCH) services in Ghana, Bangladesh, Vietnam and Rwanda. One review analysed non-financial access barriers identified in published literature using qualitative research methods [11]; the other in published literature using quantitative analysis of household survey data [12]. The paper provides a detailed summary of the methods used in the literature reviews and presents a synthesis of the key findings. It then considers the relative contributions of quantitative and qualitative studies to understanding non-financial access barriers in sufficient detail to develop effective strategies to overcome these barriers, and the level of the health system at which such analyses should be conducted and interventions developed and implemented.

## Methods

### Country focus

The literature reviews focused on MNCH in four countries: Bangladesh, Vietnam, Ghana and Rwanda. Their selection was based upon inclusion in an earlier UNICEF-Rockefeller Foundation project to assess national health insurance in Africa and Asia [13], which noted the importance of non-financial barriers to reduced access to health interventions. Each country was seen to have sufficient data sets including nationally standardised health and expenditure surveys and literature on health service access. In each country, UNICEF supports the use of Multi-Cluster Indicator Survey (MICS), Demographic and Health Survey (DHS) and various standardised household expenditure surveys that provide key sources of data. The Rockefeller Foundation also includes these four countries in their Transforming Health Systems (THS) initiative to advance an equity-focused approach to strengthening national and sub-national health systems.

### Structured literature reviews

Building on the platform of meta-ethnography, the reviews used thematic synthesis [14] so as to conduct reviews that addressed questions relating to intervention need, appropriateness, acceptability and effectiveness, without compromising the key principles developed in systematic reviews [15]. A statistical meta-analysis seeks to locate every relevant study since failure to do so may undermine the statistical models that underpin the analysis, thereby biasing the results. In meta-ethnography and thematic synthesis, however, it is not necessary to locate all available studies, but to search iteratively [14]. As Dixon-Woods et al. [16] conclude, “rather than aiming for … identification and inclusion of all relevant literature, as would be required under conventional systematic review methodology, we saw the purpose of the searching phase as identifying potentially relevant papers to provide a sampling framework”. As ‘saturation of findings’ is used in primary qualitative research, so ‘conceptual saturation’ can be used in thematic synthesis, because the purpose is interpretative explanation rather than prediction. Thematic synthesis focuses on the development of concepts and theory rather than a meticulous summary of all data [17–19]. We therefore devised ‘structured’ literature reviews, derived from the methodology for systematic reviews with rigorous study selection and defined inclusion and exclusion criteria, but without seeking to incorporate every paper published [20]. Rather, both reviews focused on being a comprehensive corpus of literature inclusive of all themes that could have been identified. Links to the structured reviews are provided in the web annex to this paper.

### Review of qualitative studies

#### Literature search and selection

The search strategy sought to identify studies that addressed non-financial barriers to access for MNCH services in the four countries. We searched for studies that included qualitative data derived from a range of methodologies including in-depth interviews, focus group discussions and survey-based studies with open-ended responses. All electronic searches used keywords covering the main search domains and a refined search string per country was used as the basic search tool for PubMed and ProQuest (incorporating the International Bibliography of the Social Sciences (IBSS); Applied Social Sciences Index and Abstracts; American Economic Association’s electronic bibliography (EconLit); and Sociological Abstracts). We used a five-phase search and inclusion/exclusion strategy.

Phase 1: The initial search included three parameters: the date of publication was 1 January 2000 to 31 December 2012; the language was restricted to English (search terms in both UK and US English were used); and the publication type in PubMed was ‘journal article’ and in ProQuest was ‘peer reviewed scholarly journal’. Results from the searches in PubMed and ProQuest resulted in 3,643 articles.

Phase 2: Two researchers independently analysed each paper by title and abstract and attributed a score: 2 for definite inclusion; 1 for potential inclusion; 0 for definite exclusion. The scores per article were added together and 262 articles totalling 4, 3 and 2 points were included in the next round. Thus 3,381 articles were excluded because they failed to satisfy one or more of the selection criteria: they were not in English; they did not focus on one or more of the four target countries; they were not qualitative; or they were not on-topic.

Phase 3: The full texts of the remaining 262 articles were reviewed for inclusion by two researchers. Any inconsistencies were discussed and resolved, and inclusion agreed through consensus. A further 174 papers were removed after the texts had been reviewed because: they were off-topic (103 papers); quantitative (60 papers); or methodologically inappropriate (11 papers). In addition, eight papers were discounted because the full text could not be accessed despite attempts to contact the primary author. The remaining 80 articles were carried forward for detailed review.

Phase 4: The full texts of the remaining 80 articles were read in detail in preparation for data extraction. During this phase the team decided to focus on two tracer interventions: skilled delivery and immunisation. Articles relating to other aspects of MNCH (breastfeeding, infant nutrition, prevention of mother-to-child transmission, etc.) were excluded (12 papers). A further 15 papers were discounted because they were found to be off-topic or predominantly quantitative. Finally, 48 papers were included in the full analysis.

Phase 5: The bibliographies of all included articles were searched for additional references, including those that might present contrary views. UNICEF country offices were contacted and a number of universities and schools of public health in each country, but these sources did not yield significant new material. A very small selection of grey literature was identified, but due to time limitations it was decided to focus the structured review entirely on scholarly articles published in peer-reviewed journals. No new literature was added at this stage.

#### Appraisal

A customised appraisal form was developed drawing on several previously published tools for qualitative research and equity [21–23]. Appraisal was undertaken in order to understand each study on its own terms, including potential bias [24], with the intention to exclude any study in which the depth and breadth of the reported data were insufficient to suggest that the findings were trustworthy [25]. No study was excluded due to assessment of conduct (validity and robustness), assessment of reporting (transparency) or assessment of content and utility of findings [21].

#### Data extraction and analysis

48 papers were analysed in full (18 focusing on Bangladesh; 19 focusing on Ghana; 11 focusing on Vietnam; and 0 focusing on Rwanda). Line-by-line coding was applied to each paper included in the review. As each subsequent paper was coded, the emerging trends were grouped together, clustering like with like and creating new codes when required. The second phase of analysis was to analyse the grouped trends and build thematic constructs per country. In this way comparisons were made within and across similar studies. In the final stage of analysis, the thematic constructs per country were synthesised into analytic themes articulating the overarching barriers to access for MNCH services. Each analytic theme contained various sub-themes, under which in-country specifics were clustered for comparison and contrast. The lead researcher for the qualitative review (JB) undertook the coding and organisation of themes by hand and no software programme was used. A second researcher then analysed the papers deductively, going back through the material and labelling each identified thematic construct as it appeared. Any inconsistencies or areas for further analysis were highlighted and discussed by the research team until all the material was resolved into appropriate themes. Through thematic synthesis of the literature reviewed, six analytical themes were identified that articulate non-financial barriers preventing access to MNCH services in the focus countries: i) perception of the condition; ii) home management and local treatment; iii) influence of family and community; iv) lack of autonomy and agency to act; v) physical accessibility; and vi) health facility and biomedical deterrents.

#### Limitations

Several authors have commented on difficulties associated with locating qualitative studies, mainly because of the poor indexing of qualitative research [26, 27]. We attempted to mitigate this limitation by supplementing the database searches with citation searching in retrieved papers, generic web searches and directly consulting experts. Although this process did not yield any new or significant material, it is possible that relevant articles may still have been inadvertently omitted. The initial search parameters, particularly restricting the language to English, may also have prevented the identification of relevant papers. This is likely to have contributed to the lack of papers from Rwanda, as a proportion of the literature focusing on MNCH in that country is published in French. Importantly, however, because of the methods employed, the synthesis of themes is comprehensive for Bangladesh, Ghana and Vietnam. Retaining the context of the research is an important aspect of synthesising qualitative literature. In our review, we were aware of the obvious contextual differences between Bangladesh, Ghana and Vietnam, but also the contextual differences found within each country. Including characteristics of the study population (e.g. their socio-economic status, ethnicity, religion, geographic location etc.) were important facets of the review and were particularly relevant in the secondary phase of the project, when the results were combined with those of the quantitative review (see below). Unfortunately, however, not every paper adequately reported the context of the qualitative research, nor the impact of the context on findings. Where possible, we systematically recorded the various contextual factors and highlighted the remaining gaps in reporting. Whilst direct findings are not necessarily generalisable, theoretical insights arising from the synthesis of the included studies should be transferrable to other similar settings.

### Review of quantitative studies using household survey data

#### Literature search

The search strategy was designed to identify studies that used household survey data to analyse and quantify non-financial barriers to access for MNCH services in the four focus countries. The literature search was conducted through online databases: PubMed; EconLit; PsycInfo; Sociological Abstracts; and the IBSS. The initial inclusion criteria stipulated that: the publication language was English; the study was conducted in one of the focus countries; the publication reported quantitative data (derived from surveys or comparable sources); the paper was published in a peer-reviewed academic journal, book or publicly available report (e.g. postgraduate dissertation); and the date of publication was between 1st January 2000 and 31st December 2012. These criteria constituted the basis of the search strings used for each database. Key terms such as ‘cross sectional’ and ‘healthcare surveys’ were embedded in the search to better carve out the applicability of existing survey data within the context of the study. The search strings were refined for each source depending on the database requirements.

#### Literature selection

A three-step screening method identified the relevant publications. Studies were excluded if: (i) an inappropriate methodology had been used (e.g. a predominantly qualitative study); (ii) if the research focused exclusively on finance, financial access barriers or health insurance; and (iii) if the main focus was not on a health service.

Step 1: Papers from the initial database searches were combined and duplicates were excluded. 1,092 were taken forward.

Step 2: Two researchers independently analysed the 1,092 papers by title and abstract. The researchers graded every publication against five criteria as strong inclusion (5 of 5), weak inclusion (4 of 5) or (3 or less) exclusion, and each were discussed until consensus was achieved. This excluded 1,009 articles.

Step 3: The full text of the remaining 83 publications was reviewed for eligibility and inclusion agreed by consensus between researchers, with 36 articles carried forward for detailed review. Snowballing and forward citation did not yield any additional studies.

#### Appraisal

All 36 studies remaining were well conducted with a methodological approach rated as either high or acceptable quality, characterised by the presentation of a well-defined underlying model or conceptual framework plus methodological rigor that acknowledged limitations and biases. No study was removed during the appraisal phase.

#### Data extraction and findings

Data extraction from the 36 eligible papers employed a tabular synopsis to consolidate relevant information. The tables included for each paper: author; year of publication; type of health service in focus; target population; and the main study question. Study design information included the data and methodology used, data sources (type and name of survey, date of survey, study population) and the specific analytic approach used. A second table for each focus country included the types of barriers identified or the different determinants of treatment seeking behaviour reported by each study. Findings were categorised according to the determinants of health services utilisation based on individual, household and community characteristics and referred to ‘predisposing’, ‘enabling’ and ‘need’ factors, following Andersen and Newman [28].

#### Limitations

The majority of the included 36 research papers analysed subsets of data from large multipurpose surveys, such as DHS. The description of specific methodological approaches and the accommodation of survey design features influencing estimation and inference were often too brief to assess the relative impact of different determinants of utilisation across different studies. Between the studies there was a significant variation of research objectives implying a risk that the individual study focus was obscured in the joint assessment as the findings were subsumed under alternate study questions.

As a variable, access to health services cannot be measured directly. Although uptake is often used as a proxy for access, access does not always translate into service utilisation [9]. Aday and Andersen [29] characterise utilisation (along with satisfaction) as an ‘outcome indicator’ of access. Neither the underlying need, nor the degree of autonomy in the decision underlying utilisation, is satisfactorily captured by survey variables. Further, the barrier itself, as a manifestation of the lack of fit between the underlying need and the service, is not reflected in household survey variables that only measure uptake. Rather, these variables, conceptualised as broad predisposing and enabling factors of healthcare utilisation, describe particular aspects of the socio-economic and socio-cultural background that promote barriers, and thus only provide one side of the access paradigm. In reviewing studies using household survey data, we adopted a pragmatic approach based on the understanding that access precedes utilisation.

The multivariate analyses of individual and community associated factors as they relate to service utilisation imply a focus on ‘determinants’ rather than ‘barriers’, even if there is a straightforward inverse relationship between the two concepts. Distinguishing different categories of determinants, such as ‘predisposing’ and ‘enabling’ factors, added complexity to the interpretation of findings, particularly if the assignment of factors to categories was ambiguous across studies. The interpretation of all variables in the respective survey requires a good understanding of the context of service provision, and the interpretation of a particular variable may change with context, e.g. the significance of education in rural vs. urban settings.

Another limitation was the substantial time lag between data collection and publication in many of the publications, which may reduce the potential relevance of survey analysis in a health policy context. Finally, the literature search was restricted to English which may have prevented the identification of relevant papers in local languages.

### Synthesis of findings

At the conclusion of both literature reviews, we (JB, MT) undertook a conjoint iterative analysis that aimed to synthesise the key qualitative and quantitative findings. Using a similar method to that developed for the review of qualitative studies, key findings were thematically analysed, highlighting points of complementarities and contradictions between the qualitative and quantitative data. This was not a reductionist exercise, rather the synthesis aimed to produce a more detailed and textured analysis than was possible by using either qualitative or quantitative data alone. This demonstrates the value of integrating qualitative and quantitative data collection and analysis to achieve an optimal mixed-methods approach from the outset, rather than synthesising the data retrospectively.

#### Limitations

The limitations outlined above for the qualitative and quantitative reviews remained valid in the synthesis of findings, which also exposed the risks of information loss and misinterpretation. The two reviews addressed fundamentally different approaches to tackle similar research questions, and the findings could not simply be matched. Whilst the qualitative studies generally elicited barriers to access, the quantitative studies emphasised factors that promoted access or were predictors of access, depending on how the survey variables were typically framed. This difference in perspective resulted in findings being formulated or presented in different ways. In the synthesis, definitions had to be refined and cross-checks conducted to ensure that like was being compared with like.

## Results

Our synthesis resulted in five dominant emerging themes that cut across both the qualitative and quantitative data: ethnicity; religion; physical accessibility; decision-making, gender and autonomy; and knowledge, information and education.

### Ethnicity

In the quantitative analysis, ethnicity emerged as an important predictor of treatment-seeking, particularly in Bangladesh and Vietnam. In the Chittagong Hill Tracts of Bangladesh, the probability of a person of non-Bengali ethnicity (Bangali, Chakma, Marma, Mru or Tripura) seeking care was significantly lower than a person of Bengali ethnicity [30]. There, the impact of ethnicity on health-seeking behaviour was found to be more pronounced than that of gender or distance to the point of service delivery. Similar findings were evident in the qualitative analysis. In discussing determinants of ante- and post-natal care seeking among the Mru, the indigenous community living in the uplands of Bandarban district, Islam and Odland [31] conclude that Mru women did not utilise health facilities even if their household was in close proximity to a facility.

With regards to household data from Vietnam, quantitative analysis concluded that ethnicity was a highly relevant determinant of health service uptake [32]. Mothers who were Kinh (the majority ethnic group) were more than twice as likely to seek care for children with diarrhoea than mothers of ethnic minorities [33]; professionally attended births were more likely if the mother was Kinh than any other ethnicity [34]; Kinh women were nearly four times more likely than ethnic minorities to give birth at health facilities [35]; and women from ethnic minorities were more likely to attend a commune health centre for delivery than Kinh women [36]. In contrast to the other studies included in the quantitative analysis, only Sepehri et al. [35] found no significant effect of ethnicity on prenatal use once other individual, household and commune characteristics had been accounted for.

The analysis of qualitative studies provided further evidence of the low utilisation of maternal health services by ethnic minorities in Vietnam and described in detail the stigma and marginalisation women experienced when seeking care [37]. It was frequently reported, for example, that most health staff were Kinh and could rarely speak local minority languages [37, 38]. Rheinländer et al. [39] observed that, “to avoid being misunderstood or perceived as backwards, [patients from ethnic minorities] never shared ideas about causes of diseases, asked clarifying questions about the prescribed drugs or told any health staff … about home-made treatments”. Both patients and health staff were frustrated that they could not communicate effectively and were forced to use over-simplistic language, and this exacerbated ethnic tensions, with a negative impact on treatment seeking by ethnic minorities.

### Religion

A number of quantitative studies across the target countries highlighted that religious affiliation could constitute a significant factor determining health service uptake. Quantitative research in Bangladesh showed differences in health service utilisation between religious groups. Young et al. [40] demonstrated that obtaining health care was positively related to being a Muslim man and negatively related to being a Hindu, and that male Hindus at every age used services less than Muslims. Rahman et al. [41] indicated a negative association between Muslim faith and the use of reproductive health services in the context of intimate partner violence.

None of the qualitative literature on Bangladesh explicitly discussed religion or faith as a barrier to treatment-seeking, but some provided important insight into local contexts. In discussing the Mru, the indigenous minority in Bandarban District, for example, Islam and Odland [31] explain that if neither the ‘traditional village healer’ or ‘village doctor’ could treat a woman’s obstetric problem, they, “pray to *Thurai* – the Mru’s god – and wait for the death of the mother”. When to seek care and what type of care to seek was morally justified by respondents who put their faith in divine intervention, and for some, illness resulting in death was regarded as inevitable and part of a divine plan or the will of God or Allah [42, 43].

The respondents’ religious background as a predisposing factor is also clear in Ghana, as shown in the multivariate analysis of the 1993 Ghana Demographic and Health Survey (GDHS) conducted by Addai [44]. It should be noted that this survey was conducted ten years before the introduction of the National Health Insurance Scheme (NHIS) in Ghana. The analysis distinguished between different types of maternal health services: prenatal care (provided by a doctor or non-doctor); antenatal care (antenatal check-up 0–3 times, or more than 3 times, for last birth); place of delivery (medical facility or home); and family planning (use of any contraceptive method). Women who adhered to ‘traditional beliefs’ tended to use prenatal care and antenatal check-ups significantly less and were far less likely to give birth in an institutional setting than members of other religions. With respect to predisposing factors to the uptake of skilled birth attendance, analyses of GDHS 2003 and 2008 also showed the relevance of religious beliefs in maternal health service utilisation: women adhering to traditional beliefs made the least use of maternal health services in Ghana [45–47]. Even after controlling for socio-economic variables, results from the GDHS 2003 indicated that Christian women were more likely to deliver at a health facility and use antenatal care more frequently than women of other religions, and that women adhering to traditional beliefs made the least use of maternal health services in Ghana [46].

Qualitative analyses largely confirmed and substantiated the household survey analyses and many of the qualitative studies from Ghana and Bangladesh emphasised the importance of faith and spirituality in treatment-seeking. Seeking care from local religious or spiritual healers was imbued with particular and significant value, and several papers concluded that, “mothers felt a spiritual gain in using them” [42]. In their study on help-seeking behaviour by childbearing women in the Ashanti Region of Ghana, Farnes et al. [48] reported that all their participants were involved with faith healing and that there was substantial overlap between Christianity, African religions and Islam. With regard to health, well-being and illness, spirituality was harnessed for both preventative (and protectionist) and curative reasons. Farnes et al. [48] concluded, however, that faith healing may, “pose problems if women rely exclusively on their faith healers and do not seek appropriate biomedical care when there are potentially serious complications”. The importance of faith in Ghana was emphasised by the use of prayer at health facilities. Clinic staff frequently incorporated prayer in their service, and accompanying relatives and friends often used the waiting room to pray for the patient [49].

In Vietnam, membership of a religious group was identified in household survey analysis to be a predisposing factor for health service uptake [34]. In their qualitative study, Rheinländer et al. [39] report that Red Dao (highland people) always sought spiritual treatment before or in parallel with treatment from the community health centre. This was seen to be ‘compatible treatment’ for children affected by angry ghosts or spells from discontent ancestors, but such factors had to be eliminated in order to make the child susceptible to biomedical treatment. Aside from localised examples, however, religion was not found to play a significant role in the Vietnam literature, due in part to dominant Communist and Confucian ideologies [50].

### Physical accessibility

The distance between a mother’s residence and the nearest health facility constitutes a key determinant of health service utilisation. This is reflected across all studies based on household survey analysis and is highlighted by the differences in service utilisation between urban and rural populations. More sophisticated spatial analysis becomes possible if a survey contains detailed geographical information, e.g. using data from geographic information systems (GIS). GIS data are increasingly available in routine household surveys (e.g. as a component of DHS surveys in over 45 countries), and are analysed and described in studies of health service utilisation and access. However, none of the studies from Bangladesh, Ghana, Rwanda and Vietnam covered by the quantitative literature review undertook any detailed analysis of geographic data, although different studies distinguished certain regional characteristics. For example, using two successive rounds of the GDHS 1998 and 2003 Amoako Johnson et al. [51] found spatial variations in the use of delivery care services at the national level, even though more than half of all births continued to occur at home without skilled obstetric care. The variations occurred within rural and urban settings across Ghana’s three ecological zones (Savannah, Forest, Coastal). Differences in service uptake by regions were not pronounced. Results indicated that barriers played a more pronounced role at lower geographical levels and may be specific to the local context [44].

Physical accessibility is a complex phenomenon and the degree to which it prevents women from using maternal and child health services depends on several conditional factors including: availability and cost of transport; road infrastructure; topographical characteristics (such as mountains or rivers); and socio-economic and socio-cultural factors closely linked to geographic location. In some analyses, physical accessibility was regarded primarily as a financial barrier, due to both the direct and indirect costs involved with journeying to a health facility (hiring transport, time spent travelling, absence from livelihood etc.) We suggest that physical accessibility should also be considered a non-financial barrier with respect to the availability of transport and the ability to travel, both logistically and socially (discussed further below). The complexity of factors underlying distance as a barrier to access are not fully apparent in quantitative research, yet in many qualitative studies, transport was seen to be a key link between the potential and actual use of services, and delays in access to care were accounted for, in part, by the lack of readily available transport. Many of the qualitative studies suggested that long distances from a patient’s home to the point of service provision was detrimental to care-seeking, particularly for routine care such as antenatal care [52]. Several reported respondents’ concern that children would become increasingly ill or die *en route* to a health facility [53], or that labouring mothers would have to give birth at the roadside. In the highlands of Vietnam, for example, there was limited transport available and some communities regarded the half or full days’ walk to the nearest community health centre to be too time consuming if the child was seriously ill and needed care quickly [39]. Edmonds et al. [54] suggest a lack of transport, “may bias patients towards using more easily accessible but potentially less trained providers,” but caregivers in the study by Rheinländer et al. [39] reported borrowing and/or spending their own money to pay for transport to travel to the clinic in a timely fashion, despite having very low incomes. As White et al. [37] emphasise, the concept of distance and ‘remoteness’ is relative, for minority families in highland Vietnam routinely travel long distances to pursue their livelihoods.

In their narrative accounts of care-seeking, respondents often referred to their transport options as ‘poor’, ‘unreliable’, ‘unsafe’ and ‘difficult to find’ and travelling at night was particularly problematic. The qualitative studies often made a distinction between emergency transport that was required suddenly for acute conditions, and the presumed ability of a family or community to make arrangements in advance (including transport arrangements in birth preparedness plans, for example). Respondents themselves were less likely to articulate this distinction and securing a means of transport and/or the finances to pay for it were rarely considered prior to their immediate need.

Modes of transport discussed include bicycles, rickshaws, pushcarts, three-wheel mini-taxis, motorbikes, boats and walking. Yet, even when transport was available, difficult terrain and the lack of roads, particularly in rural areas, often prohibited access. In some areas, levels of accessibility were seasonal and affected by the harvest and planting seasons, particularly if women were a major part of the labour force, as in Vietnam [55, 56]. In all three countries included in the qualitative review, accessibility was further curtailed during the raining season, when floodwater and mud made routes impassable [38, 54, 57, 58].

Travelling to a distant health facility is not only concerned with geographic movement, but also with social movement. Evidence from Ghana indicates that women were prepared to travel longer distances to deliver at health facilities in closer proximity to relations who could offer post-delivery assistance [59]. In Bangladesh, women were socially unable to travel alone, so unless a male relative could accompany them, seeking care at a health facility was unlikely. This suggests that even if other components of physical accessibility were in place (transport, necessary finances, passable road network etc.) culturally prescribed behaviour took precedence. Similarly, patients had to be prepared to navigate the social distance between themselves as care-seeker and the health facility as care-provider (discussed further below).

### Decision-making, gender and autonomy

Quantitative studies are unable to provide clarity on socio-cultural and socio-economic patterns promoting decision-making and provide little insight into the interplay of factors at the household level. Although a general awareness of different patterns of decision-making is reflected in the literature, quantitative analyses rarely address the interactions, although in Bangladesh, analysis did indicate that the individual characteristics of a mother should not be regarded separately from household characteristics [60, 61]. This speaks to the level of a woman’s autonomy in relation to care-seeking and decision-making and suggests that the household context may have greater significance on determining the action adopted than the mother’s characteristics alone. This finding is supported by the qualitative analysis that emphasises the ways in which decision-making about care-seeking and utilisation is a complex paradigm. Whilst it is not always well understood, it is clear that decisions are rarely taken in isolation and depend upon intra-household relations and the extended social network in which a large number of individuals play a role.

In Bangladesh, where structural components of the paternalistic society limit a mother’s agency to act [42, 43, 57] quantitative analysis of household survey data by Senarath and Gunawardena [62] showed that autonomy increased with age, level of education, participation in paid employment, and with the number of living children (see also [41, 60, 61, 63]). Education levels and paid employment were not explicitly addressed in many of the qualitative studies, yet their findings do corroborate that autonomy and influence increase with both age and number of children successfully delivered (as seen in the socially important positions of mother-in-law, female elder and grand-multiparous women) (compare [52] and [64] with [56] and [50]).

In Ghana, analysis of the 1998 and 2003 GDHS in the Ashanti Region demonstrated that despite their greater need, women utilised health services less than men [65]. The perceived quality of services impacted more on men’s uptake than on women’s, but income levels had a greater impact on women’s utilisation than men’s. The reasons for this cannot be fully explained from the quantitative analysis, but a qualitative reading of these findings suggests that if male care-seeking is required, it is likely to be given greater priority within a household’s economy than that of female care-seeking, and consequently when men seek care, they can afford to be more discerning about the quality of that care than women. Quantitative analysis indicated that in Bangladesh, girls were far less likely to be taken to a doctor (or formal professional provider) than boys [66], and there was higher likelihood that boys would benefit from health services during illness [63, 67]. Gender discrimination in relation to care-seeking for children was not raised in the qualitative studies in Bangladesh, although mothers who needed emergency Caesarean sections did report that they felt ‘lucky’ the child was a boy so that money had been spent on a son and the family were not indebted for the sake of a girl [68]. Gender issues were also raised in relation to personal interactions with health workers. In Bangladesh, social restrictions often prevent women from receiving care from male doctors [69] and many studies detailed the perception that it was ‘shameful’ for a woman to be examined by a male health worker [43, 57, 70]. Similarly, in Vietnam, women reported they felt ashamed to be attended by a male health worker [38]. In Ghana, problems appeared magnified when seeking care from male nurses, and often contributed to mothers delaying or discontinuing care [71].

In all three countries included in the qualitative review, a woman’s husband and mother-in-law (the child’s father and paternal grandmother) were regarded as particularly influential in determining the course of action. Several studies from Bangladesh concluded that, “the attitudes of in-laws and other family members delayed mothers in seeking care from clinics” [72] and there were numerous examples of families forbidding women from seeking ante- and post-natal care. Although the reasons for this were not well explained, some attributed it to a concern about violating *purdah* (the religious and social institution of female seclusion) [70, 72, 73]. Perceptions about leaving the home and minimising contact with strangers were not absolute, but could potentially contribute to prevention of or delay in access to formal services [74]. In contrast, local treatment providers were seen to be part of trusted social networks, and women in Bangladesh were able to visit certain local healers without contravening *purdah* and their restricted movements [42].

In general, care-seeking outside the home or local environments required permission from the household head in Bangladesh, Ghana and Vietnam, as it could incur both social and financial expenditure. In their study of child death in Dhaka slums, Caldwell et al. [42] state that if no male relative was available, then the mother-in-law was likely to counsel delay until they could be consulted, and in just over half of all cases of child death, inability to contact the father was recorded. Similarly, in Ghana, it was seen to be the prerogative of males and seniors to decide on treatment, as they had ‘social ownership’ of the child [64]. Although it had been assumed that husbands and in-laws negatively advised mothers who did not practice optimal care-seeking behaviour, Dearden et al. [53] found that in Vietnam, influential relations were more likely to fail to advise mothers about health than they were to provide negative advice, and at certain times, the impact of family and community may actually be an enabler that encourages service uptake [58, 72, 75].

### Education, knowledge and information

Information is a crucial enabler of health service uptake. Studies across the target countries illustrate contextual examples of the role of health knowledge as an enabling factor. It becomes obvious that education facilitates both the absorption of information and the conversion of information into knowledge. Education therefore constitutes an important criterion for the empowerment of individuals to make informed decisions on health service utilisation.

Neither of the literature reviews identified qualitative or quantitative studies that attempted a systematic approach to the analysis of information, knowledge and education, yet several reported findings about information, knowledge and education as important determinants of access. All household surveys captured the education level of the respondent. In Bangladesh, all analyses showed a positive relationship between education level and attendance at a health facility, not only of the respondent or prospective patient, but also in the household context. The level of education of a household head was a significant predictor of seeking general care [30, 63]. In Ghana, there was also a positive relationship between a woman’s level of formal education and health knowledge [76]. Each additional year of formal education significantly increased the predicted probability of use of antenatal care, skilled birth attendance and full immunisation of the child. Information was seen to be a crucial enabler of health service uptake, however lack of information about health and illness and the availability of services appears to have been assigned greater significance in the quantitative studies.

Another Ghanaian study highlighted the role and level of education as a significant predictor of physical access [77]. Lack of education was shown to dramatically reduce the probability of using maternal and child health services due to a lack of health-related information among the less educated [44]. The education of a woman’s partner was shown to influence the timing of the first antenatal care visit and the decision for skilled birth attendance [45]. An analysis of Vietnamese survey data confirmed that the higher a woman’s level of education, the more likely she was to deliver in an institutional environment [34]. A study from Bangladesh showed that husbands who understood the risk of pregnancy complications supported their wife’s use of appropriate services [61]. In the qualitative literature, education level (both individual and in a household context) was seen to be influential in terms of knowledge, decision-making and autonomy, but it was not often discussed as an independent determinate of care-seeking.

Information status depends on several characteristics of the household and its members, and information may be gained from a range of sources including the mass media or prior utilisation of health services [60]. Not surprisingly, a positive relationship exists between the information status of an individual or household and education level. Mass media exposure (that ideally conveys some rudimentary information on health and health care) positively affects the use of reproductive health services among women in Bangladesh in the context of intimate partner violence [41]. In contrast, however, Greenaway and colleagues [76] showed that in Ghana, indicators for women’s access to mass media and media exposure were not associated with use of services.

## Discussion

The synthesized findings of the literature reviews highlight a number of critical issues that have relevance for understanding and addressing access barriers in the pursuit of equitable progress to UHC. While we have explored this through the tracer of MNCH services, the issues identified are more broadly relevant. First, they highlight that there are considerable non-financial access barriers to health services; while financial barriers are important, equitable access will only be achieved if non-financial barriers are analysed and innovative policy responses developed to address them.

Second, many of the most serious non-financial access barriers relate to issues of acceptability (e.g. religious beliefs, ethnicity, autonomy). As highlighted by Gilson [78], this is the access dimension often given the least attention. Rather, the primary focus in terms of non-financial barriers is typically on availability issues (e.g. availability of appropriately trained staff, equipment and medicines). A key factor that contributes to the inadequate attention paid to acceptability barriers is that they are often seen as difficult to measure, especially relative to availability and financial access barriers. This is particularly the case where there is a reliance on quantitative methods [78]. Another factor is that addressing acceptability barriers is far more complex than those related to availability (e.g. addressing a mismatch between provider and patient attitudes and expectations compared to improving the routine availability of needed medicines).

This in turn highlights another issue; the importance of considering the bi-directional interaction between health services and the population. There remains the temptation for policy-makers and health managers to try and identify ‘supply-side’ interventions to address access barriers (e.g. to increase staffing), yet certain access barriers require more emphasis on ‘demand-side’ interventions. For example, inter-sectoral action on empowering women and engaging with opinion leaders and household decision makers may be critical in addressing social barriers that prevent the timely uptake of health services.

The findings also indicate that access barriers vary not only between countries but also between different contexts within individual countries. For example, the studies in Bangladesh and Vietnam identified specific socio-geographic areas in which factors related to ethnicity were particularly important. Similarly, certain locations may experience greater physical access barriers in comparison to others due to local topography. While national level analyses provide some insights into factors that constrain access to health services, in order to promote equitable access, detailed analyses are required at the district (or similar sub-national) level. After all, strategies for addressing both supply- and demand-side access barriers must be implemented at the district level; such strategies are unlikely to be effective if they are not tailored to the barriers specific to that district and which take account of the local context.

It is not only important that the focus of analysis be at a district/sub-national level; the nature of these analyses is equally important. Quantitative and qualitative approaches each yield unique insights, which in combination can provide information on the full range of barriers to access and use. The combined quantitative and qualitative evidence from our reviews highlights the multifaceted relationship between access barriers and across access dimensions, and how critical it is to complement quantitative indicators with qualitative data to understand the lived realities of communities as well as health care providers and provide insights into how to engage with this reality. For example, whilst quantitative methods identified disparities in services utilisation on the basis of ethnicity in Vietnam, it was qualitative studies that provided critical insights about stigma and language barriers for minority ethnic groups. This strongly suggests that mixed-methods approaches are required to diagnose access barriers within a community and identify appropriate actions to address them.

Existing qualitative and quantitative tools provide strong evidence that non-financial access barriers are real, and are relevant to explaining some important reasons for low coverage. However, there is a lack of existing frameworks to assess and prioritise financial and non-financial access barriers in a manner feasible for sub-national authorities to process and base action on. We were unable to find examples of district level approaches to systematically assess non-financial barriers to access MNCH interventions, either by qualitative or quantitative methods, in use in any of these four countries. This was in line with earlier work surveying national health insurance in Africa and Asia, which found no systematic approaches to assessing non-financial barriers to access at the national level [13].

Managers need to simultaneously identify and prioritize both non-financial as well as financial barriers, as their job is to resolve all relevant access and utilization barriers. Although there is a dearth of existing frameworks, there are, however, various District Health System Strengthening (DHSS) approaches that could be developed to incorporate a wider focus on the full range of access barriers. This would promote an ‘equity thrust’ in efforts to move towards the UHC goal of access to needed care for all through action at the sub-national or district level.

A key starting point would be to use existing data as much as possible. Although disaggregation of quantitative data can usually only happen at the regional or provincial rather than district level, national household surveys can be analysed to provide a broad indication of the categories of access barriers that are most important within that country’s context. The extent to which these categories of access barriers are of relevance in different communities can then be explored at a district level, and should be disaggregated by locally relevant population attributes to explore equity in access. Quantitative district health information system data could provide insights in this regard, yet it is important to complement this with qualitative analysis which, as our study shows, can provide important data to help operationalise the goal of promoting equity in service utilisation. It is worth noting that the districts with the greatest unmet need and facing the greatest access barriers may also be those with the weakest routine information systems and management capacity.

Our review also indicates that qualitative studies tend to be smaller, often sub-national in scale compared to quantitative studies, which limits the ability to exploit their findings more systematically. Yet, qualitative methods are not necessarily resource intensive. Innovative means of capturing community perceptions, such as electronic scorecards and the use of text-based rapid SMS technologies may provide low cost methods of gathering qualitative and quantitative data to supplement existing sources. Whether approaches such as these, or more traditional methods of community engagement are used, they should not only seek to identify the most important ‘demand-side’ barriers, but also to elicit community inputs on how best to address them and foster accountability for improving equity in service access and use. For this reason we propose assessing the feasibility of district teams using a mixed-methods approach as part of their planning and monitoring activities.

## Conclusion

There is consensus that an equitable path to UHC is desirable; such an approach prioritises the narrowing of inequalities in the access to and utilisation of health services, and ultimately of inequalities in health outcomes. In the face of increasing decentralisation of public sector management of health services the analysis of data must be disaggregated and presented in a format usable at the sub-national (often district) level, where decision making on the implementation of UHC policies and strategies generally occurs. This requires new ways of collecting data, increasing the precision of that data to track sub-national trends in access and use, and an expanded analysis of the range of factors causing barriers to access and utilisation. Vitally, this expanded diagnostic will require robust approaches that can distinguish both financial and non-financial barriers to a degree sufficient for managers to be able to take the appropriate and distinctive actions required to overcome them.

For this reason, we argue that a mixed-methods approach using qualitative and quantitative methods together to identify non-financial access barriers is required if an equitable pathway to UHC is to be implemented. Such an approach, particularly one suitable for inclusion in DHSS strategies, does not currently exist in the study countries or other countries. Previous research by the authors, combined with many years of experience working in low- and middle-income countries, suggests that neglect in identifying and addressing non-financial access barriers is widespread [13, 79]. More importantly, even within the limits of existing data, it seems quite feasible to implement a mixed-methods approach systematically into the monitoring and diagnostic components of DHSS. Future research will focus upon in-country studies to assess the feasibility of using a mixed-methods diagnostic to evaluate non-financial barriers as part of DHSS.

Our research makes a valuable contribution to discussions on UHC. It provides practical and operationally useful insights into how districts can monitor the equity impact of proposed UHC polices and strategies through expanded assessment of non-financial access barriers. This has significant implications for global and national level discussions on agreeing indicators for UHC, as we demonstrate a clear need for both qualitative and quantitative data on factors influencing access and utilisation to be collected at the district level. If every woman and every child is to count, it is imperative that they are correctly identified and that the reasons they may not use services is known and acted upon, no matter where they live or who they are.

## Additional file

Additional file 1:
**Supplement S1.** Search strings used, with links to structured reviews.
