# Supplementary material for: Synthesizing qualitative and quantitative evidence on non-financial access barriers: implications for assessment at the district level
Source: Int J Equity Health. 2015 Jun 9;14:54. doi: 10.1186/s12939-015-0181-z (PMC4467056; doi:10.1186/s12939-015-0181-z)
Supplement: Additional file 1: — Supplement S1. Search strings used, with links to structured reviews. [file 12939_2015_181_MOESM1_ESM.pdf]

Appendix

<u>Search strings qualitative review</u>

Bangladesh

((Bangladesh*) AND Health* AND (maternal OR *natal OR newborn OR infan* OR child*)) OR ((Bangladesh*) AND Health* AND (maternal OR *natal OR newborn OR infan* OR child*) AND (access* OR uptake OR utili*)) OR ((Bangladesh*) AND Health* AND (maternal OR *natal OR newborn OR infan* OR child*) AND (barrier* OR imped* OR obstacle* OR bottleneck* OR constrain*)) OR ((Bangladesh*) AND Health* AND (maternal OR *natal OR newborn OR infan* OR child*) AND (*seek* OR demand*))

Ghana

((Ghana*) AND Health* AND (maternal OR *natal OR newborn OR infan* OR child*)) OR ((Ghana*) AND Health* AND (maternal OR *natal OR newborn OR infan* OR child*) AND (access* OR uptake OR utili*)) OR ((Ghana*) AND Health* AND (maternal OR *natal OR newborn OR infan* OR child*) AND (barrier* OR imped* OR obstacle* OR bottleneck* OR constrain*)) OR ((Ghana*) AND Health* AND (maternal OR *natal OR newborn OR infan* OR child*) AND (*seek* OR demand*))

Rwanda

((Rwanda*) AND Health* AND (maternal OR *natal OR newborn OR infan* OR child*)) OR ((Rwanda*) AND Health* AND (maternal OR natal OR newborn OR infan* OR child*) AND (access* OR uptake OR utili*)) OR ((Rwanda*) AND Health* AND (maternal OR *natal OR newborn OR infan* OR child*) AND (barrier* OR imped* OR obstacle* OR bottleneck* OR constrain*)) OR ((Rwanda*) AND Health* AND (maternal OR *natal OR newborn OR infan* OR child*) AND (*seek* OR demand*))

Vietnam

(("Viet nam" OR vietnam*) AND Health* AND (maternal OR *natal OR newborn OR infan* OR child*)) OR (("Viet nam" OR vietnam*) AND Health* AND (maternal OR *natal OR newborn OR infan* OR child*) AND (access* OR uptake OR utili*)) OR (("Viet nam" OR vietnam*) AND Health* AND (maternal OR *natal OR newborn OR infan* OR child*) AND (barrier* OR imped* OR obstacle* OR bottleneck* OR constrain*)) OR (("Viet nam" OR vietnam*) AND Health* AND (maternal OR *natal OR newborn OR infan* OR child*) AND (*seek* OR demand*))

<u>Search strings quantitative review</u>

Bangladesh

((health services accessibility[MeSH Terms]) OR (utilization OR bottleneck* OR access*)) AND (survey* OR cross sectional surveys[MeSH Terms] OR health care surveys[MeSH Terms]) AND Bangladesh AND ("2000/01/01"[PDAT] : “2012/12/31"[PDAT]).

Ghana

((health services accessibility[MeSH Terms]) OR (utilization OR bottleneck* OR access*)) AND (survey* OR cross sectional surveys[MeSH Terms] OR health care surveys[MeSH Terms]) AND Ghana AND ("2000/01/01"[PDAT] : “2012/12/31"[PDAT])

Rwanda

((health services accessibility[MeSH Terms]) OR (utilization OR bottleneck* OR access*)) AND (survey* OR cross sectional surveys[MeSH Terms] OR health care surveys[MeSH Terms]) AND Rwanda AND ("2000/01/01"[PDAT] : “2012/12/31"[PDAT])

Vietnam

((health services accessibility[MeSH Terms]) OR (utilization OR bottleneck* OR access*)) AND (survey* OR cross sectional surveys[MeSH Terms] OR health care surveys[MeSH Terms]) AND Vietnam AND ("2000/01/01"[PDAT] : “2012/12/31"[PDAT])

Link to structured reviews

Access to health services: analysing non-financial barriers in Ghana, Bangladesh, Vietnam and Rwanda using qualitative methods. A review of the literature. UNICEF Working Paper. Bedford, J., A. Singh, M. Ponferrada and L. Eldred (2013).
https://www.dropbox.com/sh/1kkw1wdp10235jw/AADFJ2gYisqxa7PozHMDhwCca/2%20Anthrologica_qualitative%20methods%20non-financial%20UHC%20access%20barriers.pdf?dl=0

Access to health services: analysing non-financial barriers in Ghana, Rwanda, Bangladesh and Vietnam using household survey data. A review of the literature. UNICEF Working Paper. Thiede, M. and K. Koltermann (2013).
https://www.dropbox.com/sh/1kkw1wdp10235jw/AADQCJrgytjuc2FYiB5RiBs3a/3%20Scenarium_quantitative%20methods%20non-financial%20UHC%20access%20barriers.pdf?dl=0
